# Asymptomatic and symptomatic deep venous thrombosis in hospitalized acutely ill medical patients: risk factors and therapeutic implications

**DOI:** 10.1186/s12959-022-00433-8

**Published:** 2022-11-30

**Authors:** Lorenzo Loffredo, Gianpaolo Vidili, Angela Sciacqua, Chiara Cogliati, Rosella Di Giulio, Sciaila Bernardini, Paolo Ciacci, Antonello Pietrangelo, Federica Orlando, Aurora Paraninfi, Maria Boddi, Giovanni Di Minno, Lorenzo Falsetti, Corrado Lodigiani, Angelo Santoliquido, Evaristo Ettorre, Pasquale Pignatelli, Maria Felice Arezzo, Evghenii Gutu, Job Harenberg, Francesco Violi, AURELIO Study Group, Marco Antonio Casciaro, Sergio Morelli, Daniele Accapezzato, Elisabetta Rossi, Ilaria Maria Palumbo, Arianna Pannunzio, Alessia Fallarino, Enrico Maggio, Valeria Proietti Bocchini, Chiara Gioia, Raffaella Izzo, Raffaella Luongo, Mattia Cosenza, Maria Francesca Bisciglia, Simona Battaglia, Lohengrin Stefania Pirillo, Alessandro Capozza, Maria Luna Summa, Giuseppe Armentaro, Mara Volpentesta, Raissa Rullo, Lorenzo Baldinia, Vincenzo Arienti, Pier Luigi Meloni, Assunta Sauchella, Sara Melis, Maria Berria, Beatrice Solinas, Luca Vilardi, Paola Sarobba, Manuela Pisanu, Paolo Mangatia, Maurizio Cringoli, Deborah Blanca, Francesco Casella, Alberto Vegetti, Andrea Crociani, Emilia Donnarumma, Giulia Pacciani, Rossella Rovereto, Sarah Lunardi, Antonella Tufano, Veronica Pacetti, Marco Domenicali, Pier Leopoldo, Fabrizio Ceci

**Affiliations:** 1 Department of Clinical, Internal Medicine, Anesthesiologic and Cardiovascular Sciences, Sapienza University of Rome, Viale del Policlinico 155, 00161 Rome, Italy; 2 Department of Clinical and Experimental Medicine, University of Sassari, Sassari, Italy; 3 Department of Medical and Surgical Sciences, University Magna-Græcia of Catanzaro, Catanzaro, Italy; 4 Department of Biomedical and Clinical Sciences, L.Sacco Hospital, ASST Fatebenefratelli Sacco, University of Milan, Milan, Italy; 5 Department of Internal Medicine, Internal Medicine Unit, Maggiore Hospital, Bologna, Italy; 6 Department of Internal Medicine, AUSL Della Romagna, S. Maria Delle Croci Hospital, 48121 Ravenna, Italy; 7 Department of Internal Medicine 2, University Hospital of Modena, Modena, Italy; 8 Experimental and Clinical Department, University of Florence, Florence, Italy; 9 Department of Clinical Medicine and Surgery, Federico II University, Naples, Italy; 10 Internal and Sub-Intensive Medicine Department, A.O.U. “Ospedali Riuniti”, Ancona, Italy; 11 Cardiovascular Department, Thrombosis and Hemorrhagic Diseases Center, Humanitas Research Hospital, Rozzano, Milan Italy; 12 Catholic University of the Sacred Heart, Rome, Italy; 13 Department of Methods and Models for Economics, Territory and Finance, Sapienza University of Rome, Via del Castro Laurenziano, 9, 00161 Rome, Italy; 14 3Rd Department of General Surgery, Nicolae Testemitanu State University of Medicine and Pharmacy, Chisinau, Republic of Moldova, Chisinau, Republic of Moldova; 15 Ruprecht-Kalrs University Heidelberg, Heidelberg, Germany

**Keywords:** Deep venous thrombosis, Asymptomatic deep venous thrombosis, Compression ultrasound, Anticoagulants, Medical patient

## Abstract

**Background:**

Acutely ill medical patients experience deep venous thrombosis (DVT) during the hospitalization, however the time course of DVT is still unclear.

**Objectives:**

To evaluate risk factors in acutely ill hospitalized medical patients for proximal asymptomatic DVT (ADVT) and symptomatic DVT (SDVT) at admission and discharge.

**Patients/Methods:**

In this prospective observational study, consecutive acutely ill medical patients (hospitalized mainly for acute medical disease as infections, neoplasm, anemia, heart failure) underwent compression ultrasonography (CUS) of proximal lower limb veins within 48 h from admission and at discharge to diagnose ADVT and SDVT. Covid-19 patients, anticoagulant therapy, surgical procedures, acute SDVT, and acute pulmonary embolism, were exclusion criteria. Biographical characteristics at hospitalization, D-Dimer (assessed by ELISA)) and DD-improve score.

**Results:**

Of 2,100 patients (1002 females, 998 males, age 71 ± 16 years) 58 (2.7%) had proximal ADVT at admission. Logistic regression analysis showed that age, and active cancer were independently associated with ADVT at admission. The median length of hospitalization was 10 days [interquartile range: 6–15]. During the hospital stay, 6 patients (0.3%) with a negative CUS at admission experienced DVT (2 SDVT and 4 ADVT). In the subgroup of patients (*n* = 1118), in whom D-dimer was measured at admission, D-Dimer and IMPROVE-DD score were associated with ADVT at admission (*n* = 37) and with all DVT (*n* = 42) at discharge. ROC curve defined an IMPROVE-DD score of 2.5 as the optimal cut-off for discriminating patients with and without thrombotic events.

**Conclusions:**

We provide evidence of early development of ADVT in unselected acutely ill medical patients suggesting the need of investigating patients by CUS immediately after hospital admission (within 48 h). Advanced age, active cancer, known thrombophilia and increased IMPROVE-DD score may identify patients at risk. The benefit of anticoagulation needs to be investigated in patients with these specific risk factors and negative CUS at admission.

**Trial registration:**

NCT03157843.

## Introduction

In the last two decades, several observational and interventional studies documented that acutely ill medical patients are associated with an enhanced risk of venous thrombosis and that the use of prophylactic doses of anticoagulants, overall low molecular weight heparin (LMHH) can reduce such risk [1]. Despite the prophylaxis with LMWH have been recommended by international guidelines to lower the risk of thrombosis in this setting [2, 3], the perception of the thrombotic risk seems to be low as documented by several observational studies where LMWH prophylaxis was under prescribed [4, 5]. Such discrepancy is difficult to explain and it is also of concern the fact that the beneficial effects reported by the interventional trials are not so evident in the real-world observational studies including unselected population [5]. An important caveat of this topic is the still undefined patients’ category who would benefit from anticoagulant prophylaxis and the unclear appreciation of the real impact of hospitalization in the venous thrombosis occurrence [4, 5]. Thus, previous studies assessing the incidence of venous thrombosis in acutely ill medical patients performed a CUS after approximately 10 days from hospitalization, thereby not excluding the presence of asymptomatic venous thrombosis at admission [6, 7]. This issue has been raised by our group reporting that in acutely ill medical patients most thrombose s are detectable within 48 h from hospital admission suggesting that chronic or acute illnesses contribute to development of ADVT and SDVT before hospitalization; in this preliminary report, however, small sample size as well as incomplete definition of clinical and demographic characteristics of at risk patients limited the conclusions [8]. Due to the important questions raised by this issue including the appropriate choice of anticoagulant dosage (prophylaxis versus full anticoagulation) we performed an observational prospective study where incidences of thrombosis at admission and at discharge as well as predictors of thrombosis were examined.

## Material and methods

Two thousand one hundred consecutive non-selected patients with acute medical conditions of any kind requiring hospitalization in the internal medicine departments of the participating centers were recruited from February 2015 to July 2021. Ten centers associated to the ultrasound Study Group of the Italian Society of Internal Medicine participated in this study.

### Inclusion and exclusion criteria

As previously reported [8], to be enrolled medical patients had to be hospitalized at least 5 days. Reasons for exclusion were treatment with anticoagulant therapy at admission, surgical procedures 4 weeks before or during hospitalization, treatment with vitamin k inhibitors or direct oral anticoagulants, acute symptomatic deep venous thrombosis and acute pulmonary embolism at admission, COVID-19 (assessed by nasopharyngeal swab). Each center was advised to follow the local standard anticoagulant prophylactic management of acutely ill medical patients.

Reduced mobility was defined as requiring total bed rest or being sedentary with bathroom privileges for at least 3 days (Ref) [9]. Biographic data and comorbidities of patient were documented on admission. During the study hospitalization: heart failure was defined according to the 2013 ACCF/AHA Guideline for the Management of Heart Failure [10]; syncope, myocardial infarction and stroke were defined as previously reported [11–13]; respiratory failure was defined as a syndrome in which the respiratory system fails with hypoxemic or hypercapnic conditions; sepsis was defined according to the definition of ACCP [14], COPD was defined according to the Global Initiative for Chronic Obstructive Lung Disease (GOLD) [15]. Inherited thrombophilia was defined as known diagnosis of factor V Leiden and prothrombin G20210A mutations, presence of protein S, C or antithrombin deficiencies and antiphospholipid syndrome (APS). APS was defined according previously reported criteria (association of at least one clinical criterion (thrombosis or pregnancy morbidity) and one laboratory criterion (lupus anticoagulant (LAC), anticardiolipin antibodies (aCL) or beta2-glycoprotein I antibodies (aβ2GPI)) [16].

Compression ultrasonography (CUS) and color Doppler ultrasonography were performed within 48 h of hospitalization and before discharge (performed on the last day of hospitalization) of patients and interpreted by internists with adequate experience. Ultrasonography was routinely used to verify the diagnosis of venous thrombosis in all participating enters; no specific training for the study was necessary.

The index test was a compression ultrasonography (CUS) performed by participating MD with ultrasound machine equipped with a 7.5–10 MHz linear-array transducer and a venous vascular software. For obese subjects, a 3.5 MHz curvilinear transducer was available. Color Doppler imaging assisted vessel identification.

CUS was performed within 48 h after admission to hospital according to a standardized protocol as previously described [17]. After identification of the common femoral artery and vein located just inferior to inguinal ligament, pressure was applied until common femoral vein was completely compressed; superficial femoral vein and popliteal vein were identified and examined as a common femoral vein. Visualization of intraluminal thrombosis with incomplete compressibility of any target vein, despite adequate pressure, rendered an examination positive [17]. Examinations demonstrating complete compressibility of all target veins were considered negative [17]. CUS was performed at admission and discharge in all patients.

IMPROVE-DD score [18] and D-Dimer were assessed in a subgroup of patients. D-dimer was assessed by prevalently by an immunoturbidimetric INNOVANCE® D-Dimer Assay SIEMENS Healthiners (Italy).

The score included age > 60 years (1 point), previous VTE (3 points), known thrombophilia (2 points), current lower-limb paralysis (2 points), current cancer (2 points), immobilization ≥ 7 days (1 point), ICU/CCU stay (1 point), D-dimer ≥ 2 × the upper limit of normal (2 points).

The primary outcomes were the cumulative incidence and the clinical risk factors of proximal ADVT at admission and AVDT and SDVT at discharge.

All procedures performed in this study were in accordance with the ethical guidelines of the 1975 Declaration of Helsinki; the study was approved by the Ethical Committee of participating centers and was registered on ClinicalTrials.gov (identifier NCT03157843).

### Statistical analysis

Continuous variables are reported as mean ± SD unless otherwise indicated. Association between categorical variables was assessed by means of chi-square test. The Kolmogorov–Smirnov test was used to determine whether variables were normally distributed. Differences between groups were analyzed by Kruskal–Wallis tests for continuous variables (for non-normally distributed data) or analysis of variance (ANOVA). Univariate and multivariate logistic regression analysis was performed using a forward selection procedure. Stochastic level of entry into the model was set at a *p*-value = 0.10, and interaction terms were explored for all the variables in the final model. *P* < 0.05 was considered as statistically significant. All analyses were carried out with SPSS V.18.0 (SPSS Statistics v. 25.0, SPSS Inc. Chicago, USA).

The diagnostic performance of IMPROVE-DD score was assessed by the area under the curve (AUC) plotting receiver operating characteristic (ROC) curve that was designed to differentiate between the patients with and without deep venous thrombosis.

## Results

The entire population consisted of 2100 patients, in whom CUS was performed within 48 h from admission, 58 patients showed proximal ADVT (2.7%). All patients having ADVT at admission were immediately treated with therapeutic dosage of anticoagulants (subcutaneous LMWH enoxaparin 100 aXa IU/kg bodyweight bid followed by therapeutic dosage of anticoagulants, adjusted for body mass index (BMI) and creatinine clearance or vitamin k antagonists); 304 (14.5%) patients underwent a prophylaxis with parenteral anticoagulants (subcutaneous LWMH once daily, adjusted for BMI and creatinine clearance) at admission.

Biographic characteristics of the patients hospitalized with and without ADVT at admission are reported in Table 1. Patients with ADVT (*N* = 58) were older (77 ± 14 vs 71 ± 16 years *p* = 0.009), had a higher frequency of active cancer (29% vs 15%, *p* = 0.003), and known thrombophilia (6.9 vs 0.7%, *p* < 0.001) compared to those without ADVT (*n* = 2042) (Table 1). IMPROVE-DD score and D-dimer levels were assessed in 53% of the population (*n* = 1118) and were higher in patients with versus without ADVT (3.243 ± 1.515 vs 1.501 ± 1.442 ng/ml, *p* < 0.001). IMPROVED-D score was higher in patients with ADVT compared to those without ADVT (3.9 ± 1.7 vs 2.2 ± 1.6, *p* < 0.001).

**Table 1:** Clinical characteristics of the population with positive and negative CUS at admission. Data are reported as mean ± SD for continuous variables and % for categorical variables

	**Negative cus at admission**	**Positive cus at admission**	***P***
**N**	2042	58	-
**Age (years)**	71 ± 16	77 ± 14	**0.009**
**Age > 70 years, n (%)**	1181 (57.8%)	43 (74.1%)	**0.013**
**Female, n (%)**	971 (47.8%)	31 (53.4%)	0.399
**BMI (Kg/m**^**2**^**)**	27 ± 5	26 ± 4	0.083
**Current Smoking, n (%)**	461 (22.6%)	15 (25.9%)	0.557
**Diabetes, n (%)**	532 (26%)	12 (20.7%)	0.358
**Hypertension, n (%)**	1275 (62.4%)	34 (58.6%)	0.554
**Myocardial infarction or STROKE, n (%)**	298 (14.6%)	4 (6.9%)	0.099
**Acute infection, n (%)**	652 (31.9%)	25 (43.1%)	0.073
**Pneumonia, n (%)**	300 (14.7%)	10 (17.2%)	0.589
**Active Cancer, n (%)**	306 (15%)	15 (29%)	**0.003**
**Previous VTE, n (%)**	78 (3.8%)	5 (8.6%)	0.064
**Reduced mobility, n (%)**	549 (26.9%)	21 (36.2%)	0.115
**Thrombophilia, n (%)**	15 (0.7%)	4 (6.9%)	** < 0.0001**
**Kidney failure, n (%)**	399 (19.5%)	9 (15.5%)	0.445
**Heart or respiratory failure, n (%)**	470 (23%)	19 (32.8%)	0.083
**Dyslipidemia, n (%)**	682 (33.4%)	14 (24%)	0.140
**D-dimer (µg/mL)**^a^	1,51 ± 1,47	3,25 ± 1,49	** < 0.0001**
**Albumin (g/L)**	40 ± 9	35 ± 5	0.245
**Hormone therapy, n (%)**^b^	81 (4%)	3 (5.2%)	0.644
**Antiplatelet therapy, n (%)**^c^	796 (39%)	26 (44.8%)	0.373

^a^Performed only in patients where D-Dimer has been evaluated

^b^oral contraceptives and hormone replacement therapy (progestogen and oestrogen)

^c^acetylsalicylic acid, clopidogrel and ticagrelor

**Fig. 1:**
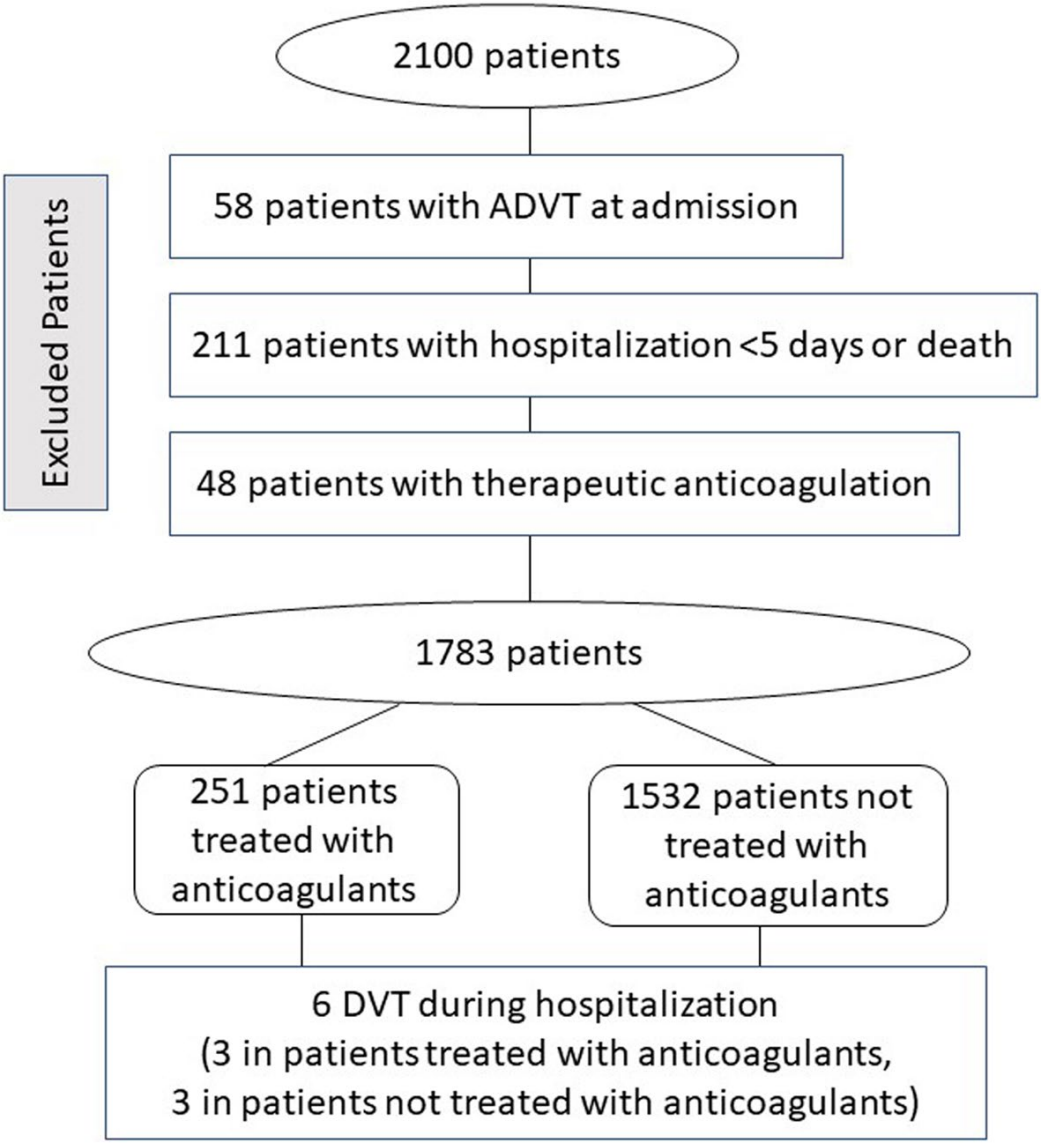
Flow chart of patients included into the study

During the intra-hospital stay 317 patients were excluded from the analysis for several reasons: 1) hospitalization < 5 days (*n* = 211) 2), thrombosis at admission (*n* = 58; 2.8%) 3) patients needing full anticoagulation for reasons other than ADVT (*n* = 48; 2.3%) (Fig. 1). During the follow-up of this remaining population (*n* = 1783), 251 (14%) patients underwent a prophylaxis with parenteral anticoagulants; the remaining 1,532 (86%) were not treated with anticoagulants (Fig. 1). The median length of hospitalization was 10 days [interquartile range: 6–15].

Clinical characteristics of the patients treated or not with an anticoagulant prophylaxis are depicted in the Table 2. Patients treated with anticoagulant were older, with a higher incidence of reduced mobility, acute infection, kidney failure, heart or respiratory failure, previous myocardial infarction or stroke and hypertension (Table 2).

**Table 2:** Clinical characteristics of patients with and without LMWH prophylaxis. Data are reported as mean ± SD for continuous variables and % for categorical variables

	**Patients With Lmwh Prophylaxis**	**Patients Without Lmwh Prophylaxis**	***P***
**N**	251	1532	-
**Age (years)**	77 ± 13	70 ± 16	** < 0.001**
**Age > 70 years, n (%)**	184 (73%)	866 (56%)	** < 0.001**
**Female, n (%)**	130 (52%)	710 (46%)	0.110
**BMI (Kg/m**^**2**^**)**	26 ± 5	27 ± 5	0.23
**Current Smoking, n (%)**	54 (21%)	339 (22%)	0.824
**Diabetes, n (%)**	68 (7%)	391 (25%)	0.598
**Hypertension, n (%)**	173 (69%)	945 (62)	**0.028**
**Myocardial infarction or STROKE, n (%)**	53 (21%)	215 (14%)	**0.004**
**Acute infection, n (%)**	103 (41%)	477 (31%)	**0.002**
**Pneumonia, n (%)**	69 (27%)	194 (13%)	** < 0.001**
**Active cancer, n (%)**	48 (19%)	221 (14%)	0.054
**History of VTE, n (%)**	15 (6%)	52 (3.4%)	0.127
**Reduced mobility, n (%)**	178 (70%)	328 (21%)	** < 0.001**
**Thrombophilia, n (%)**	2 (0.7%)	12 (0.7%)	0.982
**Kidney failure, n (%)**	66 (26%)	294 (19%)	**0.009**
**Heart or respiratory failure, n (%)**	91 (36%)	330 (21%)	** < 0.001**
**D-dimer (µg/mL)**^a^	1,93 ± 1,50	1,45 ± 1,39	** < 0.0001**

^a^Performed only in patients where D-Dimer has been evaluated

During the hospital stay, 6 patients (0.4%) experienced 4 asymptomatic and 2 symptomatic DVT; of these 3 were treated with anticoagulant prophylaxis. Two patients had heart and respiratory failure, two cancers (lung and colorectal cancer), 1 sepsis and one Moschcowitz syndrome.

Clinical characteristics of patients with and without intra-hospital DVT are depicted in Table 3. Patients with intra-hospital DVT were older and had a higher incidence of neoplasm and of thrombophilia, a lower BMI and a higher IMPROVE-DD score.

**Table 3:** Clinical characteristics of patients with positive and negative CUS during hospitalization. Data are reported as mean ± SD for continuous variables and % for categorical variables

	**Negative Proximal Cus**	**Positive Proximal Cus**	***P***
**N**	1776	64	-
**Age (years)**	71 ± 16	75 ± 13	**0.03**
**Age > 70 years, n (%)**	1046 (59%)	46 (71,9%)	**0.038**
**Female, n (%)**	928 (52%)	31 (48%)	0.515
**BMI (kg/m**^**2**^**)**	27 ± 5	25 ± 4	**0.04**
**Current Smoking, n (%)**	392 (22%)	16 (25%)	0.581
**Diabetes, n (%)**	458 (26%)	13 (20%)	0.324
**Hypertension, n (%)**	1115 (62%)	36 (56%)	0.289
**Myocardial infarction or STROKE, n (%)**	268 (15%)	4 (6%)	0.05
**Acute infection, n (%)**	577 (32%)	27 (42%)	0.105
**Pneumonia, n (%)**	262 (15%)	11 (17%)	0.62
**Active Cancer, n (%)**	267 (15%)	19 (30%)	**0.001**
**Previous VTE, n (%)**	67 (3,7%)	5 (7.8%)	0.257
**Reduced mobility, n (%)**	501 (28%)	25 (40%)	0.06
**Thrombophilia, n (%)**	14 (0,7%)	4 (6.2%)	** < 0.0001**
**Kidney failure, n (%)**	359 (20%)	10 (15.6%)	0.368
**Heart or respiratory failure, n (%)**	419 (23%)	21 (32.8%)	0.09
**IMPROVE-DD**^a^	2.3 + 1.6	3.9 + 1.6	** < 0.0001**
**D-dimer (µg/mL)**^a^	1,52 ± 1,425	3,28 ± 1,39	** < 0.0001**

^a^Performed only in patients where D-Dimer has been evaluated

In the entire population a logistic regression analysis showed that the variables associated with ADVT at admission were higher age, thrombophilia and active cancer (Table 4, Panel A). Similar data were obtained when we included the patients who developed DVT (at admission and during the intrahospital stay) with age, thrombophilia and active cancer (Table 4, Panel B).

**Table 4:** Logistic regression analysis of the variables associated with ADVT at admission (Table 4, Panel A), with DVT at admission and during the intrahospital stay (Table 4, Panel B). Logistic regression in the subgroup of patients with D-dimer analysis associated with ADVT at admission (Table 4, Panel C) and at admission and during the intrahospital stay (Table 4, Panel D)

Logistic regression analysis			
Panel A			
**ADVT at admission**			
*Variables*	*O.R*	*95% C.I*	*p*
Age	1.03	1.007–1.05	0.008
Thrombophilia	13	4–47	< 0.001
Active cancer	2.3	1.3–4.1	0.005
Panel B			
**All DVT during the hospitalization**			
*Variables*	*O.R*	*95% C.I*	*p*
Age	1.02	1.001–1.43	0.008
Thrombophilia	13	4–47	< 0.001
Active cancer	2.49	1.3–4.7	0.005
Panel C			
**ADVT at admission**			
*Variables*	*O.R*	*95% C.I*	*p*
D-Dimer	1.75	1.36–2.2	< 0.001
IMPROVE-DD	1.3	1.01–1.601	0.04
Panel D			
**All DVT during the hospitalization**			
*Variables*	*O.R*	*95% C.I*	*p*
D-Dimer	1.9	1.5–2.4	< 0.001
IMPROVE-DD	1.3	1.06—1.653	0.04

A further logistic analysis in the subgroup of patients (*n* = 1118), in whom D-dimer was measured at admission, showed that IMPROVE-DD score and D-dimer were the only variables associated with ADVT at admission (*n* = 37) (Table 4, Panel C). Similar data were obtained when all the DVT (*n* = 42) (at admission and during the intrahospital stay) were analyzed (Table 4, Panel D).

**Fig. 2:**
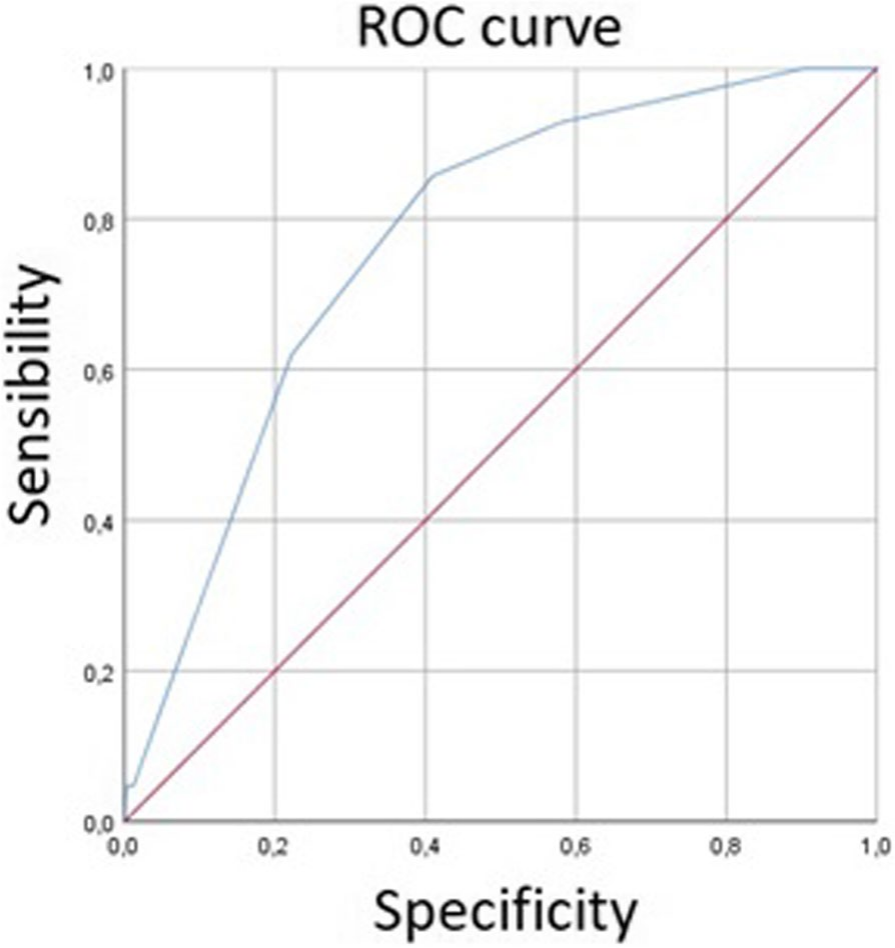
Sensitivity and specificity by IMPROVE-DD score

The optimal cut-off for discriminating patients with and without thrombotic events using the IMPROVE-DD score was 2.5 with a sensitivity of 0.85 a specificity of 0.41 (Fig. 2).

## Discussion

In this unselected population affected by acutely ill medical diseases we confirm that the incidence of proximal ADVT is > 2.5% with a clinical presentation indicating that most DVT are asymptomatic and detectable just after 48 h from admission. Conversely, the intrahospital occurrence of DVT is low with an incidence rate of 0.4%.

We have previously reported that in acutely ill medical patients an early presentation of proximal ADVT can be detected but a relatively small sample size precluded definite conclusions [8]. The present study confirms our previous report indicating that a large number of ADVT can be detected in acutely ill medical patients just after hospital admission; of note, the incidence rate is consistent with a previous study, where, however, the diagnostic work-up for ADVT was foreseen roughly 10 days from hospital admission [19]. Age, active cancer and thrombophilia were independent predictors of proximal ADVT; however, in a subgroup of patients in whom D-dimer was performed, this variable and an elevated IMPROVE-DD score were associated with proximal ADVT as reported earlier [18].

Compared to previous studies on this topic our report is peculiar for several reasons. First, we included consecutive acutely ill medical patients without any a priori selection, which better features the incidence of DVT in the real word of this setting. Second, we planned two investigations using CUS, at admission and at discharge of patients, which allowed us to better appreciate the role of hospitalization in the DVT occurrence. Thus, our data suggest that impact of hospitalization per se in the DVT occurrence is scarce, which is in contrast with the hypothesis of previous studies on this setting. Furthermore, the fact that DVT is already present just after the admission suggests that the clinical illness causing hospitalization, more than hospitalization, is likely to play a major role. The consequence of this arguments is that the guidelines on anticoagulant prophylaxis in acutely ill medical patients should be revised in order to better define the profile risk of DVT.

The study has implications and limitations. The fact that acutely ill medical patients may have developed an ADVT before entry into hospital, indicating that screening of severely ill patients upon hospitalization by CUS for detection of ADVT may be considered as a novel diagnostic and therapeutic work-up to optimize anticoagulant treatment. Thus, patients with documented ADVT should be immediately treated with full anticoagulation to reduce the DVT sequelae and eventually reduce the still elevated mortality risk (11%) occurring with the present diagnostic and therapeutic procedures [19]. In this context, the IMPROVE-DD score may be useful to identify at risk patients but further study with larger sample size is needed to confirm our results as D-dimer was not performed in all population. The number of patients with DVT during hospitalization seems to be very low suggesting a redefinition of anticoagulation prophylaxis in this setting. A limitation of the study is the low sensibility and specificity of CUS to differentiate acute from chronic DVT in patients with a previous DVT [20]. The error associated with CUS measurements of residual vein diameter, thrombus echogenicity and flow appear to be considerable to differentiate acute from chronic phase in patients with previous DVT [20, 21]. We should finally acknowledge that this study has been done in Italy and included essentially a Caucasian population; therefore, our data cannot be extrapolated to other ethnic groups.

## Conclusion

**Fig. 3:**
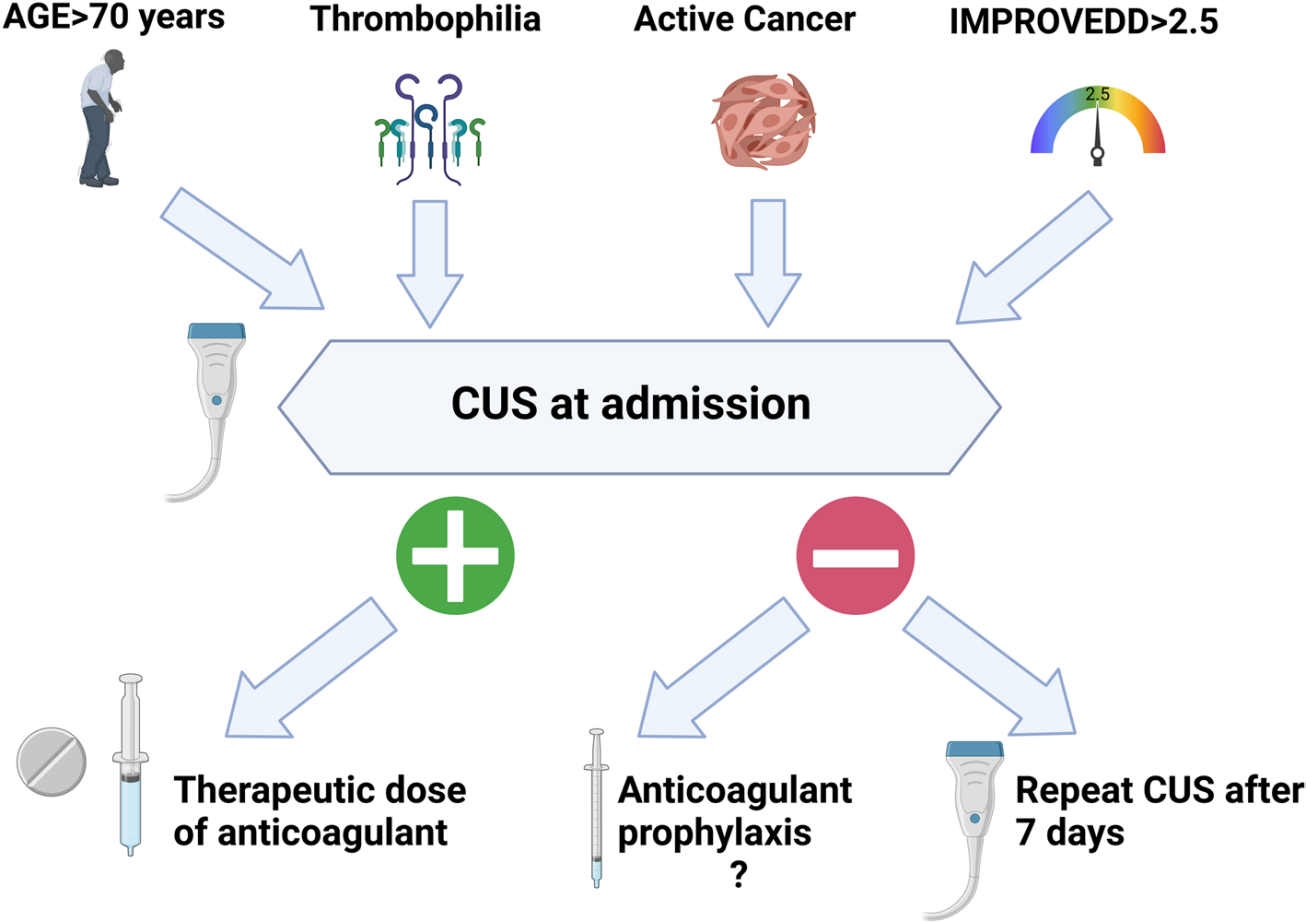
Flowchart for the diagnosis and treatment of ADVT

We provide evidence of early development of ADVT in unselected acutely ill medical patients suggesting the need of investigating patients by CUS immediately after hospital admission (within 48 h). Patients with advanced age, active cancer, known thrombophilia and increased IMPROVE-DD score should undergo CUS at admission because of higher risk of ADVT and in case of positivity be treated with therapeutic doses of an anticoagulant (Fig. 3). Taking into account the low incidence of DVT during the hospital stay, future studies should be performed to optimize anticoagulant therapy with negative CUS at admission.

## Data Availability

Data will be available upon reasonable request by Professor Lorenzo Loffredo, lorenzo.loffredo@uniroma1.it.
